# Commentary on “Pedal Medial Arterial Calcification in Diabetic Foot Ulcers: A Significant Risk Factor of Amputation and Mortality”

**DOI:** 10.1111/1753-0407.70071

**Published:** 2025-03-04

**Authors:** Mostafa Javanian, Mohammad Barary, Ali Alizadeh Khatir, Majid Khalilizad, Soheil Ebrahimpour

**Affiliations:** ^1^ Infectious Diseases and Tropical Medicine Research Center, Health Research Institute Babol University of Medical Sciences Babol Iran; ^2^ Student Research Committee, School of Medical Education and Learning Technologies Shahid Beheshti University of Medical Sciences Tehran Iran; ^3^ Department of Pharmacology, School of Medicine Shahid Beheshti University of Medical Sciences Tehran Iran; ^4^ Department of Neurology, School of Medicine Babol University of Medical Sciences Babol Iran; ^5^ Department of Orthopedics, School of Medicine Babol University of Medical Sciences Babol Iran

**Keywords:** amputation, arterial, medial, mortality, pedal, risk

Dear Editor,

We were glad to read the article entitled “Pedal Medial Arterial Calcification in Diabetic Foot Ulcers: A Significant Risk Factor of Amputation and Mortality,” published in your prestigious journal [1]. This sheds light on the yet less known association, made clear by the authors, between pedal, medial arterial calcification (MAC) and its strong relationship with amputation and mortality in diabetic foot ulcer (DFU) patients. Not only is pedal MAC a new significant predictor of amputation, but it is also a predictor of amputation independent of peripheral artery disease (PAD). However, we believe that filling in some methodological missing parts could attune the study hypothesis and strengthen the conclusions of the study.

First and foremost, one of the strengths of the study is the use of a widely recognized classification system for grading DFUs, the Wagner classification system. However, I think the study could have benefited from the inclusion of a more comprehensive ulcer scoring system, which takes into account several factors such as ulcer location, size, depth, ischemia, and neuropathy, such as the SINBAD ulcer classification [2]. This would offer a more comprehensive understanding of severity and risk, particularly for those patients with more complex presentations.

Another point of consideration is to investigate further the role of additional laboratory markers in supplementing our understanding of the clinical outcomes of the patient population. For instance, biomarkers including magnesium, zinc, vitamin B12, erythrocyte sedimentation rate (ESR), C‐reactive protein (CRP), Neutrophil‐to‐lymphocyte ratio (NLR), platelet‐to‐lymphocyte ratio (PLR), and the systemic inflammatory response index (SIRI) could provide a higher level of precision into the underlying mechanisms and may better orient the clinical risk assessment [3, 4].

The study also fails to thoroughly examine the drugs given to the patients, especially antidiabetic medicines and antibiotics. These treatments can greatly affect how well we heal and whether we have complications like amputation. A detailed breakdown of these medications and their dosages would further shed light on their potential impact on the study's findings.

Further research may also be strengthened by including additional potential comorbid conditions, including autoimmune disorders, psychiatric disorders, and cancers. It is well established that these factors have significant effects on the course of DFUs and the risk of amputation and mortality. This would give a better representation of the patient population and could potentially increase the predictive power of the model.

A deeper dive into demographic information like socioeconomic status, education level, alcohol use, and the patient's history of ulcers or amputation would also improve the study. Previous research has shown how much these variables affect health outcomes, how they would be useful in future studies, and that they should be included in reference to the totality of risk factors.

Finally, although the study conducted follow‐up telephone interviews among patients, which is informative, this methodology is based on self‐reporting. To ensure the findings are accurate and to mitigate potential bias, we suggest a more objective and structured follow‐up method.

In summary, we applaud the authors for their valuable research work, which offered crucial insight into the association between pedal MAC, the risk of amputation, and the risk of mortality in patients with diabetic foot ulcers (DFUs). We hope that the authors and the editorial board will consider these suggestions as they continue to advance research in such an important field, one that will ultimately serve our patients better and is integral to the research community.

## Author Contributions

**Mostafa Javanian:** conceptualization, investigation, supervision. **Mohammad Barary:** investigation, writing – original draft preparation, Writing – review and editing. **Ali Alizadeh Khatir:** investigation, writing – original draft preparation. **Majid Khalilizad:** investigation, writing – original draft preparation. **Soheil Ebrahimpour:** investigation, supervision, writing – original draft preparation. All authors contributed significantly to the work and approved the final version of the manuscript. Their contributions align with the latest guidelines of the International Committee of Medical Journal Editors.

## Ethics Statement

The authors have nothing to report.

## Consent

The authors have nothing to report.

## Conflicts of Interest

The authors declare no conflicts of interest.

## Data Availability

The authors have nothing to report.

## References

[1] L. Chen , D. Chen , H. Gong , et al., “Pedal Medial Arterial Calcification in Diabetic Foot Ulcers: A Significant Risk Factor of Amputation and Mortality,” Journal of Diabetes 16, no. 4 (2024): e13527, 10.1111/1753-0407.13527. 38584152 PMC10999494

[2] O. Niță , L. I. Arhire , L. Mihalache , et al., “Evaluating Classification Systems of Diabetic Foot Ulcer Severity: A 12‐Year Retrospective Study on Factors Impacting Survival,” Healthcare 11, no. 14 (2023): 2077, 10.3390/healthcare11142077. 37510519 PMC10379067

[3] S. Wang , X. Pan , B. Jia , and S. Chen , “Exploring the Correlation Between the Systemic Immune Inflammation Index (SII), Systemic Inflammatory Response Index (SIRI), and Type 2 Diabetic Retinopathy,” Diabetes, Metabolic Syndrome and Obesity 16 (2023): 3827–3836, 10.2147/DMSO.S437580. 38033457 PMC10683512

[4] J. Li , X. Wang , W. Jia , et al., “Association of the Systemic Immuno‐Inflammation Index, Neutrophil‐To‐Lymphocyte Ratio, and Platelet‐To‐Lymphocyte Ratio With Diabetic Microvascular Complications,” Frontiers in Endocrinology (Lausanne) 15 (2024): 1367376, 10.3389/fendo.2024.1367376. PMC11039910 38660516

